# The Follicular Dendritic Cells and HPV 18 Interrelation in Head and Neck Squamous Cell Carcinomas of the Larynx

**DOI:** 10.3390/medicina59061072

**Published:** 2023-06-02

**Authors:** Eugen Radu Boia, Simina Boia, Raluca Amalia Ceausu, Pusa Nela Gaje, Sarrah Mariam Maaroufi, Florica Sandru, Marius Raica

**Affiliations:** 1 Department of ENT, Faculty of Medicine, Victor Babeş University of Medicine and Pharmacy, 300041 Timişoara, Romania; eugen.boia@umft.ro; 2 Department of Periodontology, Faculty of Dental Medicine, Victor Babeş University of Medicine and Pharmacy, 300041 Timişoara, Romania; simina.boia@umft.ro; 3 Department of Microscopic Morphology/Histology, Angiogenesis Research Center, Victor Babeş University of Medicine and Pharmacy, 300041 Timişoara, Romania; pusanela@gmail.com (P.N.G.); sarrah.ma@yahoo.fr (S.M.M.); marius.raica@umft.ro (M.R.); 4 Department of Dermatovenerology, “Carol Davila” of Medicine and Pharmacy, 050474 Bucharest, Romania; florica.sandru@umfcd.ro; 5 Department of Dermatology, Elias University Emergency Hospital, 011461 Bucharest, Romania

**Keywords:** follicular dendritic cells, HPV-18, larynx, squamous cell carcinoma

## Abstract

*Background and Objectives:* Even if they are cells of controversial origin (mesenchymal, perivascular, or fibroblastic), follicular dendritic cells (FDC) are present in all organs. The aim of this study was to establish the FDC expression pattern and its interrelation with HPV 18 expression in laryngeal squamous cell carcinoma (LSCC). *Materials and Methods:* Fifty-six cases of LSCC were evaluated by simple and double immunostaining. The following score was used: 0 (negative or few positive cells), 1 (10–30% of positive cells), 2 (30–50% of cells), and 3 (over 50% of cells). *Results:* The expression of CD 21-positive cells with dendritic morphology (CDM) was noticed in the intratumoral area of conventional (well and poorly differentiated types and HPV 18 positive cases with a value of 2 for the score) and papillary types (HPV-18 negative cases with a score of 1). The highest value of 2 for the score of CDM in HPV-18 positive cases was found in the peritumoral area of well- and poorly-differentiated conventional LSCCs. A significant correlation was found between scores of CDM from the intratumoral area and those of the peritumoral area (*p* = 0.001), between CDM and non-dendritic morphology cells (NDM) of the intratumoral area (*p* = 0.001), and between HPV-18 status and peritumoral NDM cells (*p* = 0.044). *Conclusions:* The FDC and NDM cell score values of intratumoral and peritumoral areas may represent important parameters of LSCCs. This may contribute to a better stratification of laryngeal carcinoma cases and the individualized selection of clinical treatment protocols.

## 1. Introduction

Despite the medical progress that has been made in the diagnosis and therapy of larynx squamous cell carcinoma (LSCC), it remains one of the neoplasms with an increased incidence and prevalence. The following values of incidence (2.76 cases), prevalence (14.33 cases), and mortality (1.66 deaths) were described per 100,000 inhabitants and per year [1].

According to the WHO classification, the category of head and neck squamous cell carcinomas also includes those with laryngeal localization. The 4th edition of the WHO Classification of Tumours, entitled Pathology and Genetics of Head and Neck Tumours, from 2017, divided laryngeal squamous cell carcinoma into different subtypes such as conventional, verrucous, basaloid, papillary, spindle cell, adenosquamous, and lymphoepithelial SCC [2,3]. The 5th WHO Classification considered that conventional SCC is the most common tumor of the larynx, hypopharynx, and trachea, being the second most common respiratory tract cancer [4].

Among the factors that influence the prognosis and survival rate of patients with LSCC may be those of the host, tumor, and treatment. Thus, the five years survival rate for patients with early LSCC is 70 to 90%. For the patients with advanced LSCC, only a 30% for five-year survival rate was noticed [5]. In addition to risk factors such as alcohol consumption and smoking, the HPV infection was added as a pathogenic factor for LSCC. Some data suggest that HPV status may be a prognostic factor in patients with T4a LSCC [6]. About 36.9% of patients with LSCC were found to be infected with HPV-16 and -18, and integration of HPV-16 and -18 DNA into the host genome was found [7].

Another prognostic factor for tumors such as lung cancer, breast cancer, and head and neck cancer is the different degrees of tumor immune infiltration. Even if immunotherapy makes progress in the clinical area, many patients still exhibit varying degrees of toxicity and side effects after immune treatment. One of the explanations may be the immune heterogeneity of the tumors. The most important cell types revealed in the LSCC with a high immune infiltrate were activated B cells, activated CD4 T cells, activated CD8 T cells, and activated dendritic cells [8]. Therefore, analysis of the cellular profile in the intratumoral and peritumoral areas of the LSCC may be a useful stage in the development of tumor immunotherapy. One of the immunohistochemical markers that may be used for this purpose is CD 21. Its immunoexpression was found on the membrane of B lymphocytes, in the cytoplasm of some T lymphocytes and in the follicular dendritic cells [9]. Follicular dendritic cells were recognized as a central component of secondary lymphoid organs such as the spleen and lymph nodes. But their presence was also noticed in the non-encapsulated lymphoid structures. They were described in autoimmune diseases and in some neoplasias. It was launched with the idea that follicular dendritic cells (FDC) may be generated everywhere in the body. Until now, lymphoid neogenesis was mentioned in non-small lung cancer, colorectal carcinoma, ductal breast carcinoma, melanoma, lymphoma, and mucosal-associated lymphoid tissue. In the absence of B lymphocytes, FDCs are not formed [10]. The presence of B cells is correlated with increased T cell activity, which has the consequence of a better capacity of the immune system to target tumor cells and a favorable response to immunotherapy [11]. The variable immune microenvironment may influence the prognosis of HPV-positive LSCC. The clinical significance of programmed death ligand (PD-L1) in LSCC is unclear. Some data mentioned HPV status, PD-L1 expression, non-smoking status, and early stage as independent prognostic factors for LSCC patients [12].

Considering these premises, the aim of the present study was to evaluate the presence of FDC in LSCC, the interrelationship between HPV-18 expression and FDC expression, and to identify their prognostic role in squamous cell carcinomas of the larynx.

## 2. Materials and Methods

Patients and biopsies. The present study included 56 cases of larynx squamous cell carcinomas taken from hospitalized patients at the City Emergency Clinical Hospital, Timisoara, Romania, ENT Department, between 2015 and 2020. The informed consent of the patients and the Research Ethics Committee of the City Emergency Clinical Hospital Agreement Timișoara (no. 06/07.05.2018) were obtained.

Immunohistochemistry. The paraffin blocks were obtained using the TMA Grand Master System (3D Histech, Budapest, Hungary). Four cores for each tumor were taken, two from the peritumoral and two from the intratumoral areas. The morphological evaluation was followed by simple and double immunohistochemical staining. CD21 (clone 2G9) was used as the primary antibody. It was a ready-to-use antibody from Leica Biosystem Newcastle Ltd., Newcastle Upon Tyne, UK. After the pretreatment and peroxide blocking, the incubation time with the primary antibody was 20 min. The visualization was realized by using the Bond Polymer Refine Detection System. 3,3 diaminobenzidine was used as a chromogen and hematoxylin as a counterstain. For the double immunostaining of HPV18/CD21, the primary antibody (clone BF7, dilution 1:50, Novus Biologicals, Cambridge, UK, CB4 0FQ) incubation for 20 min was followed by visualization (Bond Polymer Refine Red Detection System) and chromogen application. The full immunohistochemical procedure was performed with the Bond Max Autostainer (Leica Biosystem).

Control part. The control of CD 21 immunostaining was noticed in the germinal center of the lymph node. It was noticed that cells with dendritic morphology (CDM) and membrane expression (follicular dendritic cells) and cells with non-dendritic morphology (NDM). These two positive cell types were evaluated according to the below-described score in the intratumoral and peritumoral areas of LSCC.

Microscopic evaluation and data analysis. The evaluation of the slides, picture capture, and processing were performed using a Grundium Ocus 40 microscope (Hermiankatu 6G, Tampere, Finland). In the simple and double immunohistochemical staining, the cases that showed dendritic morphology and membrane expression (CD21) and nuclear (HPV 18) were considered in the evaluation. The immunostaining results for each patient were graded as follows: 0 (negative or few positive cells), 1 (10–30% of positive cells), 2 (30–50% of cells), and 3 (over 50% of cells). The statistical evaluation was made by IBM SPSS Statistics 28, and a *p*-value of <0.05 was considered significant.

## 3. Results

The present study included 56 cases of laryngeal squamous cell carcinoma. Their morphological evaluation revealed 52 cases of conventional LSCC with the following distribution: 20 cases (well differentiated), 16 cases (moderately differentiated), and 16 cases (poorly differentiated). The adenosquamous subtype was present in two of the cases and the papillary subtype in two cases. The distribution of HPV positive cases for the conventional type was as follows: well differentiated (6 positive cases), moderately differentiated (4 of the 18 HPV-positive cases), poorly differentiated (10 cases). The LSCC subtypes were negative for HPV 18.

In the 20 cases of conventional, well-differentiated squamous cell carcinomas, the presence of keratotic and parakeratotic pearls was noted. Compared to the well- and moderately differentiated types, the sixteen cases of poorly differentiated types showed a reduction due to the absence of the previously mentioned elements and the presence of tumor cells with severe anaplastic changes. A constant feature of these cases was the presence of necrosis, variable in extent and often located in the center of the tumor areas.

The most frequent morphological pattern consisted of the identification of invasive lobules of squamous cells with intense keratinization and a variable degree of moderate to marked cellular pleomorphism. It included aspects such as a high nucleo/cytoplasmic ratio, cell apoptosis, and typical and atypical mitoses at the nuclear level noticed on the slides. Sixteen cases of moderately differentiated conventional larynx squamous cell carcinomas were characterized by the presence of large lobules of neoplastic cells with eosinophilic cytoplasm and enlarged, pleomorphic, vesicular nuclei with a hypertrophic nucleolus. The presence of keratotic pearls was noted.

The blood vessels showed small sizes, an aspect noted especially in poorly differentiated conventional cases of LSCCs. Erythrocytes and tumor emboli were observed in the blood vessel lumen. Two ways of disposing the tumor cells in the lumen were noted: free or attached to the vascular endothelium.

Two of the cases showed the disappearance of cell adhesion inside the tumor areas, a feature that led to the identification of a pseudo-glandular appearance. These morphological aspects characterize the adenosquamous type.

Two cases with finger-like stromal cores and several layers of squamous epithelium revealed a papillary type of LSCC.

The internal positive control of the CD21 reaction was confirmed by the presence of follicular dendritic cells at the level of the germinal centers of the secondary follicles. Membrane expression was also found in the lymphocytes of the germinal center. For follicular dendritic cells, a morphology with numerous extensions communicating with each other and forming a network in the germinal center of the secondary follicle was noticed.

CD21 simple immunostaining revealed for the conventional, well-differentiated LSCC a value score of 0 for CD21 expression in cells with dendritic morphology (CDM) in the tumor areas. In the peritumoral microenvironment, cells with dendritic morphology and membrane expression were quantified with a score of 2 (in 6 cases). In all these cases, the absence of cells with non-dendritic morphology (NDM) in the intratumoral and peritumoral areas (score 0) was noticed.

Conventional moderately differentiated LCSS was characterized by the absence of CDMs in the tumor area in all evaluated cases. A tendency for the positive cells to be located at the periphery of the keratotic pearls was noticed. In the peritumoral stroma, CD 21 positive CDM types were quantified with a score value of 1 in all sixteen cases. NDM cells were absent in the intratumoral area except in some cases. In the peritumoral area, the following scores were noticed: absence of NDMC cells (2 cases) and score 1 (14 cases).

Poorly differentiated conventional cases of LSCC (16 cases) were characterized by the absence of CDM in the tumor area in all cases except two. A higher score was found in the peritumoral area for CDM. Thus, the value of scores 1 (4 cases), 2 (6 cases), and 0 (6 cases) was found. The distribution of NDMs in the peritumoral area showed a score of 1 (4 cases) and 0 (12 cases). Most of these cases were characterized by the absence of NDM in the peritumoral area (score 0), except for 2 cases (score 2).

Both cases of adeno-squamous LSCC type did not present CD 21-positive NDMs in the intratumoral and peritumoral areas. CDM were found in the peritumoral area only (score 1).

In the papillary type of LSCC (2 cases), the CDM CD21-positive cells were noticed in the peritumoral area only (score 1). The NDMs were not identified.

CD21/HPV-18 double immunostaining indicated a heterogeneous distribution and score values of CDM and NCDM in the peritumoral and intratumoral areas.

All cases of moderately differentiated conventional LSCCs were characterized by a score value of 0 for CD21-positive CDM in the intratumoral area. For this cell type, the same score was noticed in the well-differentiated conventional LSCC cases, except for two of them, which were HPV-18 positive, evaluated with a score of 2. Two cases of poorly differentiated LSCCs, HPV 18 positive presented CDM (cells with dendritic morphology), CD21 positive, assessed with a score of 2, in the tumor area. Both cases of adenosquamous LSCCs had a CDM score of 0. The two cases of the papillary subtype were evaluated with a score of 1 for CD21-positive CDM in the tumor area.

CD21-positive cells with dendritic morphology (CDM) in the peritumoral area were identified. Well-differentiated conventional LSCC cases had a value of 0 for these cells score except for two HPV-18 positive cases assessed with a score of 2. A value of 1 for the score was noticed in the moderately differentiated conventional LSCCs: in HPV-18 negative cases (eight cases) and in HPV-18 positive cases (four cases). A value of 0 for the score was found in four HPV-18 negative cases. The following score values were shown in poorly differentiated conventional LSCC: a value of 0 for 10 cases; a value of 1 (4 cases) and a value of 2 (2 cases, both of them HPV-18 positive). The adeno-squamous and papillary subtypes had a value of 0 for the score. Two of the cases showed co-expression of CD21/HPV 18 in cells with dendritic morphology in the peritumoral area. These types were evaluated with a score of 0.

The highest values of CDM scores for the intratumoral and peritumoral areas in the HPV-18 positive cases of conventional LSCC are detailed in Table 1.

The adeno-squamous LSCC type was HPV-18 negative and did not present CDM or NDM in the intratumoral or peritumoral areas. Both cases of LSCC of the papillary type were HPV-negative. The highest value of CDM (score 1) was found in the intratumoral area.

**Figure 1:**
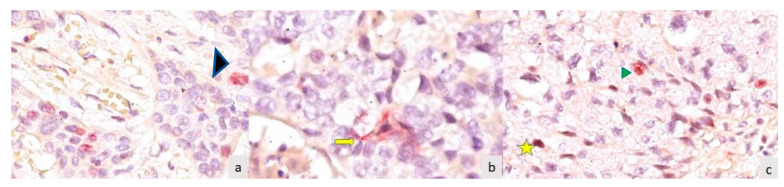
Main cell types in the CD21/HPV 18 double immunostaining (**a**) Intratumoral CD 21 positive cell with non-dendritic morphology (NDM) (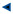
—black arrow) in a conventional LSCC, moderately differentiated, HPV negative, CD21/HPV18 double immunostaining, ×400 magnification; (**b**) conventional LSCC, moderately differentiated, HPV 18 negative, CD21 positive cells, with dendritic morphology (CDM) (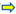
—yellow arrow), CD21/HPV18 double immunostaining, ×400 magnification; (**c**) conventional LSCC, poorly differentiated, HPV 18 positive cells (yellow star—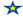), and non-dendritic morphology (NDM), CD21 positive cells (green arrow—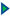), CD21/HPV18 double immunostaining, ×400 magnification.

The main cell types noticed in the conventional LSCC, moderately and poorly differentiated, are shown in Figure 1.

The analysis of the CD21-positive NDM cells in the intratumoral area of well differentiated conventional LSCCs revealed a value 0 for the score in two HPV 18 positive cases and in four HPV-18 negative cases. Twelve cases presented a value 1 for the score (two of them HPV 18 positive cases and ten of them HPV18 negative cases). Two HPV 18 positive cases were assessed with a score of 2. Value of score 1 was found in ten cases of moderately differentiated conventional LSCCs (two of them HPV18 positive and eight of them HPV-18 negative). A total of cases (2 of them HPV 18 positive and 4 of them HPV-18 negative) had a value of 0 for the score. Two cases presented co-expression of CD21/HPV 18 with a score of 0 for the NDM cells of the intratumoral area. For the poorly differentiated conventional LSCCs the following values of score were noticed: value of 0 (6 cases, two of them HPV 18 positive and four of them HPV 18 negative); value of 1 (6 cases, four of them HPV 18 positive and two of them HPV 18 negative); value of 2 (four cases, all of them HPV 18 positive). The HPV 18 negative adenosquamous type had a value of 0 for the score. The HPV-18 negative papillary type had a value of 1 for the NDM cell score of the intratumoral area.

Assessment of cells with non-denritic morphology in the peritumoral area showed a value of score of 0 for fourteen cases of well differentiated conventional LSCCs. Ten of them were HPV-18 negative and four of them HPV 18 positive. Score of 1 was also identified in the same type of LSCC in six of the cases (two of them HPV-18 positive and four HPV-18 negative). The same values 0 and 1 for the score were noticed in the moderately differentiated conventional LSCC. HPV-18 positive cases had a value 0 of score. HPV 18 negative cases showed a value of 0 for the score (four cases) and value of 1 (eight cases). Values of 0 and 1 for the NDM cell score from peritumoral area were found in poorly differentiated conventional LSCC. HPV-18 positive cases had a value of 0 for score (6 cases) and value of 1 (four cases). A total of 6 HPV-18-negative cases had a value of 0. The adeno-squamous and papillary subtypes had a value of 0 for score.

A significant correlation was found between the intratumoral score of CDM and the peritumoral one (*p* = 0.001). A representative correlation was noticed between the intratumoral score of CDM and the intratumoral score of NDM cells (*p* = 0.001). An important correlation was observed between the score of NDM cells from the peritumoral area and HPV-18 status (*p* = 0.0446). No significant correlation was found between the score of CDM and HPV-18 status.

## 4. Discussion

Squamous cell carcinoma of the larynx is responsible for 81,806 deaths in males and 12,965 in females in 2018 [13]. Most of the patients had an advanced stage of the disease at the time of diagnosis. In addition, the incidence of squamous cell carcinomas with this localization is constantly increasing.

A rate of HPV infection ranging from 3 to 85% has been demonstrated in laryngeal carcinomas. No statistically significant differences were found between the presence of HPV DNA in normal specimens and in neoplastic mucosa specimens [14]. In the case of laryngeal carcinomas, the prognostic role of HPV infection is still debated, between authors who consider HPV a positive prognostic factor and other authors who contradict this hypothesis. Some studies have noted a favorable prognosis of laryngeal carcinomas with HPV infection, with improved three-year overall survival but no significant long-term improvement [15]. In the present study, HPV-18 positivity was noted in 37.03% of cases.

Follicular dendritic cells were initially considered as cells limited to the germinal centers of lymphoid follicles and having a memsenchymal origin in the marrow. Among their roles, it was mentioned that antigen-presenting cells that stimulate the activity of helper T cells and effector B cells, which mediate humoral immune responses. It has been thought to directly influence the selection, differentiation, and proliferation of B cells [16]. Most ultrastructural studies have indicated a fibroblastic origin for FDCs in their less differentiated forms. However, unlike fibroblasts, in mature forms, follicular dendritic cells display a network of long, branching extensions, making desmosomal and adherens junctions. The idea that follicular dendritic cells can be generated anywhere in the body suggests either that their precursors have significant mobility or that they originate from a non-migratory precursor. In this sense, an experiment was carried out in a murine model. Platelet-derived growth factor receptor beta-positive cells, purified from the vascular stromal fraction of the spleen, were transplanted into the renal capsule of mice lacking endogenous follicular dendritic cells, resulting in the appearance of lymph nodes containing fully differentiated follicular dendritic cells. This experimental model supports the idea that follicular dendritic cells were generated from perivascular cells. The omnipresence of these perivascular cells and, consequently, the precursors of follicular dendritic cells explain their presence in any tissue or organ. It has not been demonstrated whether any mural cell can give rise to follicular dendritic cells or whether they must originate from specific tissues such as adipose tissue [17].

CD21, the complement receptor type 2 (CR2), belongs to the family of regulators of complement activation. It is a 145 kDa glycoprotein expressed mainly by B lymphocytes and follicular dendritic cells and to a lesser extent by thymocytes, a subpopulation of peripheral T lymphocytes, cervical and pharyngeal epithelial cells, basophils, natural killer cells, and astrocytes. It binds complement fragments iC3b, C3dg, and C3d, this receptor for EBV and HIV and is involved in B cell responses to T cell dependent antigens [18]. The expression pattern of CD 21 was membrane, mainly for the B lymphocyte and cytoplasmic for the T lymphocyte [19]. In the present study, two CD 21 positive cell types were found with dendritic morphology (CDM) and non-dendritic morphology (NDM) in the tumor and peritumoral areas. A heterogeneous pattern was also noticed.

The follicular dendritic cells and their involvement in other types of neoplasia was shown by some data. Thus, Bagdi et al. [20] noticed their role in the diagnosis of lymphoproliferative disorders. Germinal center FDCs in lymphoid hyperplasias and expanded FDC meshwork in the mantle cell lymphomas, MALT lymphomas, low-grade follicular lymphomas were intensely stained with CD21, CD23, and CD35 antigens. In our study, FDCs or CDMs with a membrane expression pattern were noticed in the germinal center of lymph node. CD21-positive cells with dendritic morphology (CDMs) and cells with non-dendritic morphology (NDM) were found in the intratumoral and peritumoral areas of LSCCs.

Immunohistochemical analyses of clinical B-cell lymphoma samples revealed CD21 expression in all evaluated cases [21]. In our study, CD21 expression was found in cells with non-dendritic morphology in the intratumoral and peritumoral areas and in the lymphocytes of the germinal center.

In the case of squamous cell carcinomas, there are numerous immune cells in the tumor microenvironment, but their functions have not been well established completely until now. There are no biomarkers yet to indicate the response of HNSCC patients to immunotherapy. The identification of immune-related genes can improve prognosis, more efficient stratification of patients, individualized selection of clinical treatment protocols.

The assessment of antigen-presenting B cells (BAPC) in blood (peripheral blood mononuclear cells, PBMC) and tumor samples of patients with cancer revealed that BAPCs were increased in the tumor microenvironment of majority analyzed cancer types with site-specific variation. A systemic increase was noticed in tumor-infiltrating lymphocytes (TIL) and PBMCs in patients with colorectal cancer and gastroesophageal adenocarcinoma. BAPCs were localized in lymphoid follicles of tertiary lymphoid structures (TLS) and were enriched in tumors with increased numbers of TLSs. BAPCs isolated from tumor-draining lymph nodes of patients with cancer showed increased percentages of tumor antigen-specific B cells and induced responses of autologous T cells in vitro [22]. In the present study, CD21 positive non-dendritic morphology cells (NDM) were found in the intratumoral and peritumoral areas of LSCCs. A significant correlation was found between the NDM cells score and cells with dendritic morphology (FDC) in the intratumoral area.

B lymphocyte composition and function in head and neck squamous cell carcinoma (HNSCC) has not been well known. Lechner et al. [18] demonstrates different immune infiltration patterns in relation to serological TAA response detection and the presence of B cell subpopulations in HNSCC. Their role could be tumor promoting and antitumor activity. They considered it will be important to include B cells into comprehensive phenotypic and functional analyzes of tumor-associated lymphocytes for a better immunotherapeutic approach. In our study a significant correlation was noticed between the HPV-18 status and NDM cells of peritumoral area.

Several mechanisms by which the tumor evades immune mechanisms have been described: reduction of antigen presentation, cytokine production, resistance to apoptosis, recruitment of regulatory T cells, myeloid-derived suppressor cells. All this favors the appearance of an immunosuppressive microenvironment, inducing tumor growth. Differences were observed between the types of immune cells infiltrating tumor cells. Thus, for HPV-positive cases, the following were noted: the ratio between inflammatory macrophages M1 and anti-inflammatory macrophages M2-positive, T cells, CD8 positive, and B cells present. HPV-negative cases presented in the tumor area: positive M1/M2 ratio, positive natural killer cells, positive myeloid dendritic cells, positive T lymphocytes, and CD8 [23]. No data were available for the B lymphocytes. In our study, which focused on the larynx area, the tumor area was identified: tumor cells, CD21 positive cells with dendritic morphology and with non-dendritic morphology. CD21-positive cells with and without dendritic morphology were also found in the peritumoral stroma in variable proportion.

In the case of laryngeal squamous cell carcinoma, the tumor immune microenvironment consists of immune cells disposed in the center of the tumor, invasive margin, or surrounding stroma. The idea was launched that the maturity of TLSs can be accurately classified by H&E staining. FL-TLS is a potential mediator of antitumor immunity in human laryngeal cancer [24]. In our study, cells with dendritic morphology were found but also cells with non-dendritic morphology and that were CD21-positive were found, which may represent different types of lymphocytes. These cells were found in the intratumoral but mainly in the peritumoral area.

## 5. Conclusions

Two types of CD21 positive cells, with dendritic (FDC) and non-dendritic morphology, were found in the LSCC in the intratumoral and peritumoral areas. These aspects may be useful for completing the heterogeneous cellular image and the mechanisms aimed at the resistance and efficiency of immunotherapy in larynx squamous cell carcinoma.

## Figures and Tables

**Table 1:** The mainly positive type cells in the CD21/HPV-18 double immunostaining.

WHO Classification Type	Intratumoral Cells with Dendritic Morphology CDM	Peritumoral Cells with Dendritic Morphology CDM	HPV 18 Positivity
Conventional, well differentiated (20 cases)	Score 2 2 cases	Score 2 2 cases	6 cases
Conventional, moderately differentiated (16 cases)	Score 0 (16 cases)	Score 1 (14 cases)	4 cases
Conventional, poorly keratinized (16 cases)	Score 2 (2 cases)	Score 2 (2 cases)	10 cases

## Data Availability

Not applicable.
